# Supplementary material for: Age-structured non-pharmaceutical interventions for optimal control of COVID-19 epidemic
Source: PLoS Comput Biol. 2021 Mar 4;17(3):e1008776. doi: 10.1371/journal.pcbi.1008776 (PMC7963091; doi:10.1371/journal.pcbi.1008776)
Supplement: S1 Text — (PDF) [file pcbi.1008776.s007.pdf]

S1 Text. Computations of the adjoint system

In order to deal with the necessary optimality conditions, we use some results in [1]. Next, we detail the computations of the adjoint system (12)-(13). To this end, we first define the functions $y_1, Q : [0,T] \times [0,a_{\max}] \to \mathbb{R}$ and $y_2 : [0,T] \times [0,a_{\max}] \times \mathbb{R}_+$ by:

$$y_1(t,a) = \begin{pmatrix} S(t,a) \\ R(t,a) \end{pmatrix}, \quad y_2(t,a,i) = \begin{pmatrix} I_s(t,a,i) \\ I_m(t,a,i) \\ I_p(t,a,i) \end{pmatrix}, \quad Q(t,a) = \begin{pmatrix} H(t) & E(t,a) & b(t,a) \end{pmatrix}$$

wherein

$$g_H(i, y_2(t,a,i)) = I_s(t,a,i) 1_{[i_{sympt},\infty)}(i), \qquad g_R(i, y_2(t,a,i)) = \sum_{k \in \{s,m,p\}} h_k(a,i) I_k(t,a,i),$$

$$g_\lambda(a,i,y_1,y_2) = S(t,a) \times$$
$$\int_0^{a_{\max}} K(a,a') \left(\beta_s(a',i) I_s(t,a',i) + \beta_m(a',i) I_m(t,a',i) + \beta_p(a',i) I_p(t,a',i)\right) \mathrm{d}a',$$

$$H(t) = \int_0^\infty \int_0^{a_{\max}} g_H(i, y_2(t,a,i)) \mathrm{d}a \, \mathrm{d}i, \qquad E(t,a) = \int_0^\infty g_\lambda(a,i,y_1(t,a,i), y_2(t,a,i)) \mathrm{d}i,$$

$$b(t,a) = \int_0^\infty g_R(i, y_2(t,a,i)) \mathrm{d}i.$$

The model (5) thus rewrites as

$$\begin{cases} \partial_t y_1(t,a) &= F_1(a, Q(t,a), c(t,a), y_1(t,a)), \\ (\partial_t + \partial_i) y_2(t,a,i) &= F_2(a,i,Q(t,a),c(t,a),y_2(t,a,i)), \\ y_2(t,a,0) &= \Phi(a, c(t,a), E(t,a)), \end{cases}$$

with

$$F_1(a,Q(t,a),c(t,a),y_1(t,a)) = \begin{pmatrix} -\mu(a,H(t))S(t,a) - (1-c(t,a))E(t,a) \\ -\mu(a,H(t))R(t,a) + b(t,a) \end{pmatrix},$$

$$F_2(a,i,Q(t,a),c(t,a),y_2(t,a,i)) = \begin{pmatrix} -(\mu(a,H(t)) + \gamma(a,i,H(t)) + h_s(a,i)) I_s(t,a,i) \\ -(\mu(a,H(t)) + h_m(a,i)) I_m(t,a,i) \\ -(\mu(a,H(t)) + h_p(a,i)) I_p(t,a,i) \end{pmatrix},$$

and

$$\Phi(a,c(t,a),Q(t,a)) = \begin{pmatrix} (1-p)q(a)(1-c(t,a))E(t,a) \\ (1-p)(1-q(a))(1-c(t,a))E(t,a) \\ p(1-c(t,a))E(t,a) \end{pmatrix}.$$

We now rewrite the functional J as

$$J(c) = \int_0^T \int_0^{a_{\max}} \left(\mathscr{J}_1(a,c(t,a),Q(t,a),y_1(t,a)) + \int_0^\infty \mathscr{J}_2(a,i,Q(t,a),y_2(t,a,i)) \mathrm{d}i \right) \mathrm{d}a \, \mathrm{d}t$$

which is decomposed into

$$\mathscr{J}_1(a,c(t,a),Q(t,a),y_1(t,a)) = \mu_{add}(a,H(t))(S(t,a)+R(t,a)) + B(a)c^2(t,a)$$

and

$$\mathscr{J}_2(a,i,Q(t,a),y_2(t,a,i)) =$$
$$\gamma(a,i,H(t))I_s(t,a,i) + \mu_{add}(a,H(t))(I_s(t,a,i)+I_m(t,a,i)+I_p(t,a,i)).$$

We denote by $z_1, \zeta_k : [0,T]\times[0,a_{\max}] \to \mathbb{R}$ (for $k \in \{1,2,3\}$) the following adjoint functions

$$z_1(t,a) = (z_S(t,a), z_R(t,a)), \quad \zeta(t,a) = (\zeta_1(t,a), \zeta_2(t,a), \zeta_3(t,a)),$$

and we denote by $z_2 : [0,T]\times[0,a_{\max}]\times\mathbb{R}_+$ the following adjoint function

$$z_2(t,a,i) = (z_{I_s}(t,a,i), z_{I_m}(t,a,i), z_{I_p}(t,a,i)),$$

satisfying $\lim_{i\to\infty} z_2(t,a,i) = 0$ and $z_1(T,a) = z_2(T,a,i) = 0$. We get

$$\nabla_{y_1}\mathscr{J}_1(a,c(t,a),Q(t,a),y_1(t,a)) = \begin{pmatrix}\mu_{add}(a,H(t))\\ \mu_{add}(a,H(t))\end{pmatrix}^T$$

$$\nabla_{y_2}\mathscr{J}_2(a,i,Q(t,a),y_2(t,a,i)) = \begin{pmatrix}\mu_{add}(a,H(t))+\gamma(a,i,H(t))\\ \mu_{add}(a,H(t))\\ \mu_{add}(a,H(t))\end{pmatrix}^T$$

$$\nabla_{y_1}F_1(a,Q(t,a),c(t,a),y_1(t,a)) = \begin{pmatrix}-\mu(a,H(t)) & 0\\ 0 & -\mu(a,H(t))\end{pmatrix}$$

and

$$\nabla_{y_2}F_2 =$$
$$\begin{pmatrix}-\mu(a,H(t))-\gamma(a,i,H(t))-h_s(a,i) & 0 & 0\\ 0 & -\mu(a,H(t))-h_m(a,i) & 0\\ 0 & 0 & -\mu(a,H(t))-h_p(a,i)\end{pmatrix},$$

where $\nabla_y F$ denotes differentiation of F with respect to the variable y.

Then

$$(z_1\cdot\nabla_{y_1}F_1)(t,a) = \begin{pmatrix}-\mu(a,H(t))z_S(t,a) & -\mu(a,H(t))z_R(t,a)\end{pmatrix}$$

and

$$(z_2\cdot\nabla_{y_2}F_2)(t,a,i) = \begin{pmatrix}-(\mu+\gamma+h_s)z_{I_s}(t,a,i) & -(\mu+h_m)z_{I_m}(t,a,i) & -(\mu+h_p)z_{I_p}(t,a,i)\end{pmatrix}.$$

Setting

$$g_1(a,y_1,y_2) = \begin{pmatrix}\int_0^\infty g_H(i,y_2(t,a,i))\mathrm{d}i\\ E(t,a)\\ b(t,a)\end{pmatrix}, \quad g_2(a,i,y_1,y_2) = \begin{pmatrix}g_H(i,y_2(t,a,i))\\ g_\lambda(a,i,y_1(t,a,i),y_2(t,a,i))\\ g_R(i,y_2(t,a,i))\end{pmatrix},$$

we see that

$$\nabla_{y_1} g_1(a,y_1,y_2) =$$
$$\begin{pmatrix} 0 & 0 \\ \int_0^\infty \int_0^{a_{\max}} K(a,a')(\beta_s(a',i)I_s(t,a',i)+\beta_m(a',i)I_m(t,a',i)+\beta_p(a',i)I_p(t,a',i))\mathrm{d}a'\ \mathrm{d}i & 0 \\ 0 & 0 \end{pmatrix}$$

and

$$\nabla_{y_2} g_2(a,i,y_1,y_2) = \begin{pmatrix} \mathbf{1}_{[i_{sympt},\infty)}(i) & 0 & 0 \\ S(t,\cdot)\beta_s(a,i)K(\cdot,a) & S(t,\cdot)\beta_m(a,i)K(\cdot,a) & S(t,\cdot)\beta_p(a,i)K(\cdot,a) \\ h_s(a,i) & h_m(a,i) & h_p(a,i) \end{pmatrix}.$$

From there, we deduce that

$$(\zeta\cdot\nabla_{y_1} g_1)(t,a) =$$
$$\begin{pmatrix} \zeta_2(t,a)\int_0^\infty \int_0^{a_{\max}} K(a,a')(\beta_s(a',i)I_s(t,a',i)+\beta_m(a',i)I_m(t,a',i)+\beta_p(a',i)I_p(t,a',i))\mathrm{d}a'\mathrm{d}i & 0 \end{pmatrix}$$

and

$$(\zeta\cdot\nabla_{y_2} g_2)(t,a,i) =$$
$$\begin{pmatrix} \zeta_1(t,a)\mathbf{1}_{[i_{sympt},\infty)}(i)+\beta_s(a,i)\int_0^{a_{\max}}\zeta_2(t,a')S(t,a')K(a',a)\mathrm{d}a'+\zeta_3(t,a)h_s(a,i) \\ \beta_m(a,i)\int_0^{a_{\max}}\zeta_2(t,a')S(t,a')K(a',a)\mathrm{d}a'+\zeta_3(t,a)h_m(a,i) \\ \beta_p(a,i)\int_0^{a_{\max}}\zeta_2(t,a')S(t,a')K(a',a)\mathrm{d}a'+\zeta_3(t,a)h_p(a,i) \end{pmatrix}^T.$$

The adjoint system is given by

$$\begin{cases} -\frac{\partial z_1}{\partial t}(t,a) &= \nabla_{y_1}\mathscr{J}_1(t,a)+(z_1\cdot\nabla_{y_1}F_1)(t,a)+(\zeta\cdot\nabla_{y_1}g_1)(t,a) \\ -(\frac{\partial z_2}{\partial t}+\frac{\partial z_2}{\partial i})(t,a,i) &= \nabla_{y_2}\mathscr{J}_2(t,a)+(z_2\cdot\nabla_{y_2}F_2)(t,a,i)+(\zeta\cdot\nabla_{y_2}g_2)(t,a,i) \end{cases}$$

which is equivalent to (12). Next, we see that

$$\nabla_Q\Phi(t,a) = \begin{pmatrix} 0 & (1-p)q(a)(1-c(t,a)) & 0 \\ 0 & (1-p)(1-q(a))(1-c(t,a)) & 0 \\ 0 & p(1-c(t,a)) & 0 \end{pmatrix}$$

whence

$$(z_2(\cdot,\cdot,0)\cdot\nabla_Q\Phi)(t,a) = \begin{pmatrix} 0 & [1-c(t,a)][(1-p)(q(a)z_{I_s}+(1-q(a))z_{I_m})+pz_{I_p}](t,a,0) & 0 \end{pmatrix}.$$

Further, we have

$$\nabla_Q\mathscr{J}_1(t,a) = \begin{pmatrix} \frac{\partial\mu}{\partial H}(a,H(t))(S(t,a)+R(t,a)) & 0 & 0 \end{pmatrix}$$

and

$$\nabla_Q\mathscr{J}_2(t,a,i) =$$
$$\begin{pmatrix} \frac{\partial\mu}{\partial H}(a,H(t))(I_s(t,a,i)+I_m(t,a,i)+I_p(t,a,i))+\frac{\partial\gamma}{\partial H}(a,i,H(t))I_s(t,a,i) & 0 & 0 \end{pmatrix}.$$

We also see that $\nabla_Q g_1 \equiv 0$, $\nabla_Q g_2 \equiv 0$,

$$\nabla_Q F_1(t,a) = \begin{pmatrix} -\frac{\partial\mu}{\partial H}(a,H(t))S(t,a) & -(1-c(t,a)) & 0 \\ -\frac{\partial\mu}{\partial H}(a,H(t))R(t,a) & 0 & 1 \end{pmatrix}$$

and

$$\nabla_Q F_2(t,a,i) = \begin{pmatrix} -\left(\frac{\partial\mu}{\partial H}(a,H(t)) + \frac{\partial\gamma}{\partial H}(a,i,H(t))\right) I_s(t,a,i) & 0 & 0 \\ -\frac{\partial\mu}{\partial H}(a,H(t))I_m(t,a,i) & 0 & 0 \\ -\frac{\partial\mu}{\partial H}(a,H(t))I_p(t,a,i) & 0 & 0 \end{pmatrix}$$

whence

$$(z_1 \cdot \nabla_Q F_1)(t,a) = \begin{pmatrix} -\frac{\partial\mu}{\partial H}(a,H(t))S(t,a)z_S(t,a) - \frac{\partial\mu}{\partial H}(a,H(t))R(t,a)z_R(t,a) \\ -(1-c(t,a))z_S(t,a) \\ z_R(t,a) \end{pmatrix}^T$$

and

$$(z_2 \cdot \nabla_Q F_2)(t,a,i) = \begin{pmatrix} -\left(\frac{\partial\mu}{\partial H} + \frac{\partial\gamma}{\partial H}\right) I_s z_{I_s} - \frac{\partial\mu}{\partial H} I_m z_{I_m} - \frac{\partial\mu}{\partial H} I_p z_{I_p} & 0 & 0 \end{pmatrix}.$$

Finally, the adjoint functions ζ must satisfy the following equation:

$$\begin{aligned} \zeta(t,a) =& (z_2(\cdot,\cdot,0) \cdot \nabla_Q \Phi)(t,a) + (\nabla_Q \mathscr{J}_1(t,a)) + (z_1 \cdot \nabla_Q F_1)(t,a) + (\zeta \cdot \nabla_Q g_1)(t,a) \\ &+ \int_0^\infty (\nabla_Q \mathscr{J}_2(t,a,i) + (z_2 \cdot \nabla_Q F_2)(t,a,i) + (\zeta \cdot \nabla_Q g_2)(t,a,i))\,\mathrm{d}i \end{aligned}$$

which is equivalent to (13). Finally by [1], the Hamiltonian is given by

$$\mathscr{H}(t,a,c) = z_2(t,a,0) \cdot \Phi(t,a,c,Q) + \mathscr{J}_1(a,c,Q,y_1) + \int_0^\infty \mathscr{J}_2(a,i,Q,y_2)\mathrm{d}i$$

which leads to

$$\begin{aligned} \mathscr{H}(t,a,c) = & E(t,a)[1-c(t,a)][(1-p)(q(a)z_{I_s} + (1-q(a))z_{I_m}) + pz_{I_p}](t,a,0) \\ &+ \mu_{add}(a,H(t))(S(t,a)+R(t,a)) + B(a)c^2(t,a) \\ &+ \int_0^\infty (\gamma(a,i,H(t))I_s(t,a,i) + \mu_{add}(a,H(t))(I_s(t,a,i) + I_m(t,a,i) + I_p(t,a,i)))\,\mathrm{d}i. \end{aligned} \quad \text{(A.1)}$$

References

[1] Feichtinger G, Tragler G, Veliov VM. Optimality Conditions for Age-Structured Control Systems. Journal of Mathematical Analysis and Applications. 2003 Dec;288(1):47–68.
