# Supplementary material for: Age-structured non-pharmaceutical interventions for optimal control of COVID-19 epidemic
Source: PLoS Comput Biol. 2021 Mar 4;17(3):e1008776. doi: 10.1371/journal.pcbi.1008776 (PMC7963091; doi:10.1371/journal.pcbi.1008776)
Supplement: S2 Text — (PDF) [file pcbi.1008776.s008.pdf]

S2 Text. Basic reproduction number

Here we compute the basic reproduction number R_0 of the model (3)-(5). First let us set for $i \geq 0$ and $a \in [0, a_{\max}]$ the following functions

$$\pi_s(a,i) = \exp\left(-i\mu_{nat}(a) - \int_0^i [\gamma_{dir}(a)\mathbf{1}_{[i_{sympt}, i^s_{\max}]}(\sigma) + h_s(a,\sigma)]\mathrm{d}\sigma\right),$$

$$\pi_m(a,i) = \exp\left(-i\mu_{nat}(a) - \int_0^i h_m(a,\sigma)\mathrm{d}\sigma\right),$$

$$\pi_p(a,i) = \exp\left(-i\mu_{nat}(a) - \int_0^i h_p(a,\sigma)\mathrm{d}\sigma\right),$$

that describe the survival probability of infected individuals (in the respective compartment), with age a, from their infection until the time since infection i, in case of no hospitalisation (*i.e.* $H \equiv 0$). We get the following Volterra formulation of the linearized system of (3)-(5):

$$I_s(t,a,i) = \begin{cases} I_{s,0}(a, i-t)\frac{\pi_s(a,i)}{\pi_s(a,i-t)}, & \text{for } t \in [0,i), \\ (1-p)q(a)\lambda_0(t-i,a)S_0(a)\pi_s(a,i), & \text{for } t \geq i, \end{cases} \tag{B.1}$$

$$I_m(t,a,i) = \begin{cases} I_{m,0}(a, i-t)\frac{\pi_m(a,i)}{\pi_m(a,i-t)}, & \text{for } t \in [0,i), \\ (1-p)(1-q(a))\lambda_0(t-i,a)S_0(a)\pi_m(a,i), & \text{for } t \geq i \end{cases} \tag{B.2}$$

and

$$I_p(t,a,i) = \begin{cases} I_{A,0}(a, i-t)\frac{\pi_p(a,i)}{\pi_p(a,i-t)}, & \text{for } t \in [0,i), \\ p\lambda_0(t-i,a)S_0(a)\pi_p(a,i), & \text{for } t \geq i \end{cases} \tag{B.3}$$

where $\lambda_0 = \lambda(\cdot,\cdot,0)$ is defined by

$$\lambda_0(t,a) = \int_0^{a_{\max}} K(a,a') \int_0^{\infty} \big(\beta_s(a',i)I_s(t,a',i) + \beta_m(a',i)I_m(t,a',i) + \beta_p(a',i)I_p(t,a',i)\big)\,\mathrm{d}i\,\mathrm{d}a', \tag{B.4}$$

where β_k, $k \in \{s,m,p\}$ are defined transmission probabilities. Let $I_N(t,a) = \lambda_0(t,a)S_0(a)$ be the density of newly infected of age a at time t, with $c \equiv 0$. Then (B.1)-(B.2)-(B.3) can be rewritten as the following Volterra formulation:

$$I_N(t,a) = S_0(a)\int_0^t \int_0^{a_{\max}} K(a,a')\omega(a',i)I_N(t-i,a')\mathrm{d}a'\,\mathrm{d}i + f(t,a),$$

where

$$\omega(a',i) = \beta_s(a',i)(1-p)q(a')\pi_s(a',i) + \beta_m(a',i)(1-p)(1-q(a'))\pi_m(a',i) + \beta_p(a',i)p\pi_p(a',i)$$

and $f(t,a)$ is the density of new infections produced by the initial population. Therefore, the basic reproduction number R_0 is the spectral radius, denoted by $r(U)$, of the next generation operator U defined on $L^1_+(0,a_{\max})$ by

$$U : L^1(0,a_{\max}) \ni v \longmapsto S_0(\cdot)\int_0^{\infty}\int_0^{a_{\max}} K(\cdot,a')\omega(a',i)v(a')\mathrm{d}a'\,\mathrm{d}i \in L^1(0,a_{\max})$$

As explained before, it is estimated in [1] that each average infectiousness β_k ($k \in \{s,m,p\}$) takes the form of a Weibull distribution $W(3,5.65)$ so that the mean and median are equal to 5.0 days while the standard deviation is 1.9 days. Based on this estimation, we assume that $\beta_k(a,i) = \alpha\overline{\beta}(i)\xi_k(i)$ where $\overline{\beta} \sim W(3,5.65)$ and α is a positive parameter to be determined. Consequently, it follows that α is given by

$$\alpha = \frac{R_0}{r\left(\overline{U}\right)}, \tag{B.5}$$

where $\overline{U}$ is the operator defined by

$$\overline{U} : L^1(0,a_{\max}) \ni v \longmapsto S_0(\cdot)\int_0^\infty \int_0^{a_{\max}} K(\cdot,a')\overline{\varpi}(a',i)v(a')\mathrm{d}a'\ \mathrm{d}i \in L^1(0,a_{\max})$$

with

$$\overline{\varpi}(a',i) = \overline{\beta}(i)\left[\xi_s(i)(1-p)q(a')\pi_s(a',i) + \xi_m(i)(1-p)(1-q(a'))\pi_m(a',i) + \xi_p(i)p\pi_p(a',i)\right].$$

We see that $\overline{U}$ can be rewritten as

$$\overline{U}v(a) = S_0(a)\int_0^{a_{\max}} K(a,a')\overline{\Omega}(a')v(a')\mathrm{d}a',\ \forall v \in L^1_+(0,a_{\max}),$$

where $\overline{\Omega}(a') = \int_0^\infty \overline{\varpi}(a',i)\mathrm{d}i$. Now, in order to compute the spectral radius $r\left(\overline{U}\right)$, we first make the following assumptions:

Assumption .1 *We suppose that functions $S_0, K, \overline{\Omega}$ are bounded and positive almost everywhere.*

Then, we can show that $r(\overline{U})$ is given by the spectral radius of the following linear operator:

$$L^1(0,a_{\max}) \ni v \longmapsto \int_0^{a_{\max}} K(\cdot,a')\overline{\Omega}(a')S_0(a')v(a')\mathrm{d}a' \in L^1(0,a_{\max})$$

which can be easily computed since the age a is numerically divided into N classes, so that the term inside the integral of the latter equation is a $N \times N$ matrix. Finally, we obtain α from (B.5).

In addition to Assumption .1, if the function K is symmetric, we can define the positive self-adjoint operator S by

$$S : L^2(0,a_{\max}) \ni v \longmapsto \sqrt{S_0(\cdot)\overline{\Omega}(\cdot)}\int_0^{a_{\max}} K(\cdot,a')\sqrt{S_0(a')\overline{\Omega}(a')}v(a')\mathrm{d}a' \in L^2_+(0,a_{\max}).$$

We then deduce the following

Proposition .2 *Let K be symmetric and Assumption .1 be satisfied. Then, operators $\overline{U}$ and S are positive and compact, their spectra $\sigma(\overline{U}) \setminus \{0\}$ and $\sigma(S) \setminus \{0\}$ are composed of isolated eigenvalues with finite algebraic multiplicity. Moreover, we have $\sigma(\overline{U}) = \sigma(S) \subset \mathbb{R}_+$ and the following Rayleigh formula holds:*

$$r(\overline{U}) = r(S) = \sup_{\substack{v \in L^2(0,a_{\max}) \\ \|v\|_{L^2(0,a_{\max})}=1}} \int_0^{a_{\max}} \int_0^{a_{\max}} K(a,a')\sqrt{S_0(a')\overline{\Omega}(a')}\sqrt{S_0(a)\overline{\Omega}(a)}v(a')v(a)\mathrm{d}a'\ \mathrm{d}a.$$

Proof. The compactness of both integral operators follows from the fact that $a_{\max} < \infty$ by assumption (see Table 1), hence their spectra are punctual. Now we prove that $\sigma(\overline{U}) = \sigma(S)$. Let $\nu \in \sigma(\overline{U})$ be an eigenvalue of $\overline{U}$ and $\phi \in L^1(0, a_{\max})$ be the associated eigenvector, *i.e.*

$$\overline{U}\phi(a) = S_0(a) \int_0^{a_{\max}} K(a,a')\overline{\Omega}(a')\phi(a')\mathrm{d}a' = \nu\phi(a), \quad \forall a \in [0, a_{\max}]$$

so that $\phi \in L^\infty(0, a_{\max}) \subset L^2(0, a_{\max})$. Defining the function

$$\psi = \frac{\phi\sqrt{\overline{\Omega}}}{\sqrt{S_0}} \in L^2(0, a_{\max})$$

leads to

$$\nu\psi(a) = \sqrt{S_0(a)\overline{\Omega}(a)} \int_0^{a_{\max}} K(a,a')\sqrt{\overline{\Omega}(a')S_0(a')}\psi(a')\mathrm{d}a' = S\psi(a), \quad \forall a \in [0, a_{\max}]$$

i.e. $\nu \in \sigma(S)$ is an eigenvalue of S associated to the eigenvector ψ, so that $\sigma(\overline{U}) \subset \sigma(S)$. For the reverse inclusion, let $\nu \in \sigma(S)$ and $\psi \in L^2(0, a_{\max}) \subset L^1(0, a_{\max})$ be the associated eigenvector for S. It follows that the function

$$\phi = \frac{\psi\sqrt{S_0}}{\sqrt{\overline{\Omega}}} \in L^1(0, a_{\max})$$

is an eigenvector of $\overline{U}$ related to the eigenvalue $\nu \in \sigma(\overline{U})$, whence $\sigma(\overline{U}) = \sigma(S)$. In particular, both spectral radius are equal. Finally, the Rayleigh formula is classical for positive and symmetric operators. ∎

References

[1] Ferretti L, Wymant C, Kendall M, Zhao L, Nurtay A, Abeler-Dörner L, et al. Quantifying SARS-CoV-2 Transmission Suggests Epidemic Control with Digital Contact Tracing. Science. 2020 Mar.
