# Supplementary material for: Age-structured non-pharmaceutical interventions for optimal control of COVID-19 epidemic
Source: PLoS Comput Biol. 2021 Mar 4;17(3):e1008776. doi: 10.1371/journal.pcbi.1008776 (PMC7963091; doi:10.1371/journal.pcbi.1008776)
Supplement: Attachment — Submitted filename: CoverLetter-QR-SA-MC-MTS-RDD-V2.pdf [file pcbi.1008776.s009.pdf]

Dear Editor,

we wish to thank you and the reviewer for the careful evaluation of our work. We read the reviews with interest and were greatly encouraged to resubmit our manuscript.

In this revised version, we have tried to address the concerns raised during the initial round of review.

In particular, we now apply our age-structured model to three countries with contrasted demography: France, Vietnam, and Burkina Faso. Generating more concrete results allows us to better illustrate the potential of the model.

We also added a sensitivity analysis to study the effect of 5 key model parameters on output variables such as total number of infections, hospital admissions, and deaths.

Furthermore, we also realised that more details were necessary to explain optimal control theory and how it differs from a classical optimisation approach. To this end, we heavily re-wrote the introduction.

Finally, all the minor issues have been corrected and more detailed answers are provided below. The initial comments are in black and our response is in blue. The changes made are further outline in italics.

We thank you again for your interest in our work and hope that our manuscript is now acceptable for publication in *PLoS Computational Biology*.

Sincerely,
On the behalf of authors,
Dr. Ramsès Djidjou-Demasse.

Reviewer: 1

The results are not surprising, especially given the number of age-dependent models that consider similar controls such as social distancing. (See some of the references below for a sub-sample.) The current paper needs to really show how certain latent times, such as time to symptomatic or asymptomatic populations, change results that are known. Sensitivity analysis is really needed here if one wants to make a case for an optimal control problem applied to standard modeling efforts has something significant to say.

Answer: One important point to stress here is that, contrary to existing age-dependent models, we develop an optimal control theory approach. In other words, we are not simply attempting to optimise a single control parameter per age class a (say x_a) but rather how this control should vary over time (so $x_a(t)$ using the same notation). We now further stress this important fact in the Introduction.
About the age structure of the host population, we agree that this is
a known key determinant of acute respiratory diseases, as stressed by the works cited by the reviewer. Regarding the importance of the age of the infection on COVID epidemic short-term dynamics, this has also been shown as in Sofonea et al (in the main text). However, models allowing for this two double continuous structure (host age and infection age) are rare, and, amongst these, we are not aware of any that is analysed in the context of optimal control theory.

↪ *We now better explain in the Introduction the originality of the framework, which is to implement an optimal control theory approach for a model with a double age structure. We also better introduce the specificity of optimal control theory for a wider audience*

Regarding global sensitivity analyses, we study how infected individuals, hospitalizations, and deaths change in response to variations in five parameters: the proportion of infections that are paucisymptomatic (p), the average time to symptoms onset (i_{sympt}), the infectiousness reduction of paucisymptomatic infections (ξ_p), the healthcare maximum capacity (H_{sat}), and the population structure (including the natural mortality, the size of the population, age-structure, and social contacts). Population structure is the main parameter highlighted by the sensitivity analysis with 70% of the variance explained for the number infected individuals and 40% for hospitalizations and deaths. This parameter is followed by ξ_p, p, and i_{sympt} which have a similar effect on the number infected individuals. By contrast, for hospitalizations and deaths, the population structure parameter is followed by p with 40% and 30% of the variance explained respectively; while ξ_p and i_{sympt} have very marginal impact. Finally, the importance of H_{sat} is strictly negligible on the three output variables, with however, a greater importance on deaths as compared to hospitalizations and infected.

↪ *We added the results of the sensitivity analysis in page 11.*

There really is no analysis of the model, other than some existence control theory. Some analysis can be done in simple models, such as Hindes et al below, and should be able to expand to age structured models.

Answer: We did analyse some of the key properties of the model and, for instance, we computed the basic reproduction number R_0 in the appendix B, defined as the spectral radius of some linear operator, and gave a practical way to derive it numerically (Proposition B.2).

↪ *See page 32-34 for more details.*

However, we indeed did not perform a full analysis for several reasons. First because the general structure of the model is itself similar to existing models (see Sofonea et al 2020 for an example). Second because the originality of the approach resides more in the application of optimal control and the double age structure and we did not want to overburden the reader (some of our analytical results are actually already in the appendix). Third, some of the results are very challenging to prove formally. For instance, we also gave some references about the well-posedness of the PDE model where two continuous structures are considered (age and time since infection in our case). Only a few papers with such double structure exist in the literature, as we mentioned in the introduction, and the well-posedness is far from trivial and cannot be addressed in few lines (see Burie *et al.* in the main text).

↪ *See page 5 for more details.*

About the reference Hindes *et al.* mentioned by the reviewer, the analysis concerns a classical SEIR

ODE-model. It neither accounts for the age structure of the population, nor social contacts (one of the key determinants of acute respiratory diseases). The computations of eigenvalues performed for such a simple model cannot easily be expanded to age structured models (PDE), where the study of the spectra of linear operators are much more challenging. For additional details on this issue, see *e.g.* G.F. Webb (1985): *Theory of Nonlinear Age-dependent population dynamics* or K.J. Engel and R. Nagel (2000): *One-parameter semigroups for linear evolution equations.*

A key assumption is highly questionable: the infectiousness is assumed to be independent of age. This needs to be altered in multiple ways - asymptomatic, young people being the most likely superspreaders.
Answer: On the biological side, the review's claim of young people being superspreader seems difficult to back up with current evidence. In fact, although superpsreading events have been documented, there is still much uncertainty about their relative role in the spread of the epidemic and about their origin (superspreading could be linked to environmental conditions, such as massive gatherings, rather than individual properties). Therefore, assuming independence from age seems the most parsimonious assumption given the current data.
↪ *We mention this fact in the manuscript (see page 20).*
From a modelling point of view, assumption that the infectiousness is assumed to not depend on host age is only made for parameterization purpose, based on estimates in (Ferretti et al) which is independent of age. This assumption does not impact the general formulation of the model proposed here. However, the progression of infection to stages severely infected, mildly infected and paucysymptomatic is determined by the proportion of paucysymptomatic (p) and symptomatic requiring hospitalization (q). While q is age dependent and estimated by (Ferguson et al), p is assume variable and is now included in our global sensitivity analysis.
↪ *See equation (3) for the incoming flow of each group and section on the sensitivity analysis.*

Table of parameters: Where is the latency in the equations of motion? If it is a strict delay, should the model be an integral- delay equation? Same with the time to infection onset. Is it assumed to be exponential?
Answer: Contrarily to classical SEAIR models, disease-stage progression in our model is not captured by discrete compartments (exposed, asymptomatic, and infected) with exponentially distributed waiting times to switch between compartments. The advantage of our formalism is that disease progression can be modelled using a continuous variable, called the time since infection (in days) denoted by i. Every infected person then remains in the "infection" compartment from exposure until recovery (or death). The time since infection grows linearly with time, according to the derivative with respect to i. Latency from exposed to asymptomatic and time of symptoms onset are not needed for this modelling approach because these are captured through the functions describing the transmission rate, the mortality rate, and the recovery rate at time i post infection.
More precisely, the average latency from exposed to asymptomatic (i_{lat}) is simply mentioned to define the average time to infection onset (i_{sympt}), and also to help the readers to understand the model flow diagram (Figure 1).
On the other hand, the time to infection onset (i_{sympt}) is used to define the infectiousness reduction factors (ξ_s, ξ_m) and the mortality rate due to the infection (γ). Note also that the parameter $\overline{\beta}$ used to define the transmission probabilities β_s, β_m and β_p (see Section 3.2), is computed in Ferretti *et al.* by using, among other, this time to infection onset.
↪ *We clarified this in the manuscript (see remark at page 3)*

Fig.4: All controlled peaks are above capacity. They are reduced, but still high. Also, number of deaths are reduced, which is expected. But how much is due to hospitals having better experience in time and more equipment as time moves on. A lot of hospitals are operating below capacity and have greatly improved the death rate. This should be reflected in the prediction and definitely is missing from the model.
Answer: The reviewer is correct in that some of the external parameters may vary with time. As

the saying goes, “all models are wrong, but some are usefull”. All models are wrong because they will never capture the reality with all its details and we must make choices in what to consider. The model proposed here does not take into account the adapting hospital capacity to meet changing demands during the epidemic course. However, the global sensitivity analysis shows that the importance of this parameter is marginal compared to population structure (including age-structure and social contacts), the proportion of paucisymptomatic or the infectiousness reduction of paucisymptomatic.

Reviewer: 2

My main “concern” with this manuscript is that the authors write as if they were applying Pontryagin's maximum principle to a system of ODE's (something very common) while they are working on a PDE model (which is less common, and more challenging). I understood that one can similarly derive a Hamiltonian, express the optimal control and solve the state and adjoint system using e.g. a forward-backward sweep method. However, I was not able to follow the construction of the adjoint system in appendix based on the references provided (which are likely the most relevant ones though). I would have appreciated a more didactic appendix but this is obviously not a relevant request in this context.
Answer: We thank the reviewer for this clear and constructive concern. We have tried to bring more didactic and details on the derivation of the adjoint system in the Introduction so that a general audience can better grasp the originality and challenge of the approach.

My second possible ”concern” has to do with the reproducibility of the results. It is unclear to me whether the code will be provided as an online resource. I realize that the authors use a WAIFM matrix from a previous study but it would be good to make the code publicly available if not yet done (especially for a journal like PLoS Comp. Biol.).
Answer: Indeed, the code will be provided as an online resource, as suggested.
↪ *We heartily agree with this concern and apologize for not being more thorough in this issue in our inital version. The code (with the Julia Programming Language) used to simulate the model can now be accessed through the Zenodo platform http://doi.org/10.5281/zenodo.4288144*

Guest Editor: Gabor Balazsi

Could some altered cost functions be tested, trying to include more than just deaths? Long-term hospitalizations and long-term health consequences would be worth somehow factoring in. It is unclear whether these additional age-dependent costs would be proportional to the number of deaths.
Answer: Other objective functions can be considered including for example, long-term hospitalizations and long-term health consequences. It is equivalent to considering the number of hospitalizations as the variable to be minimized and costs associate to long-term hospitalizations and long-term health consequences. This formulation may indeed be interesting to look at in details but would deserve to be considered independently in another study.
↪ *We mention this fact in the Discussion (see page 19).*

there should be a perspective on long-term consequences. In particular, how many infected individuals remain in the population? What is expected over 2 years, 3 years, 10 years with various control strategies? How soon would a new wave emerge and what would be the costs?? Could the future costs be discussed or maybe included?
Answer: More details are now provided to on the proportion of infected individual at the end of the control period. The average proportion of infected individual is then compared to the herd immunity threshold $(1 - 1/R_0)$ for each control measure considered.
↪ *See section Results.*
