# Supplementary material for: Age-structured non-pharmaceutical interventions for optimal control of COVID-19 epidemic
Source: PLoS Comput Biol. 2021 Mar 4;17(3):e1008776. doi: 10.1371/journal.pcbi.1008776 (PMC7963091; doi:10.1371/journal.pcbi.1008776)
Supplement: Attachment — Submitted filename: CoverLetter-QR-SA-MC-MTS-RDD-V2-R1.pdf [file pcbi.1008776.s010.pdf]

Dear Editor,

please find attached a revised version of our manuscript entitled *Age-structured non-pharmaceutical interventions for optimal control of COVID-19 epidemic.*

First, we would like to again thank the anonymous reviewer for his/her careful reading of our manuscript.

We have incorporated the suggestions made, which we think increase the clarity and focus of our manuscript. In the following, we provide details answers to the comments made.

We also read the text carefully to improve the writing and remove typoes.

We thank you again for your interest in our work and hope that our manuscript is now acceptable for publication.

Yours sincerely,
On the behalf of authors,
Dr. Ramsès Djidjou-Demasse.

Reviewer: 1

Author summary. I do not have exactly the same interpretation as the authors regarding France and Vietnam, for which the optimal strategy seems to be composed of two different periods:
1/ The first part of the optimal strategy consists in protecting the most vulnerable people (60+ years old), and if the cost is intermediate, to protect 40/50+ people as well (Fig. 6 and S3), which helps controlling the epidemic (temporarily). Indeed the epidemic restarts at the end of the control period if the cost is low or intermediate, while it does not restart if the cost is high. In other words, if the cost is high, the only thing one can do is to protect older people during the uncontrolled epidemic, which lasts a hundred days.
2/ The second part of the optimal strategy targets epidemic control rather than the protection of older people. After an initial period of a hundred days, the number of cases is below the health care capacity, and the optimal control applies to age classes within the interval [40,60] if the cost is low or intermediate. Strikingly, the optimal control does not target 60+ people any longer. This is particularly clear in Vietnam (Fig. S3). The same trend applies to France (Fig. 6). In particular, the optimal control is zero for the 80+ age-classes. The optimal strategy in Burkina Faso is composed of only the first part: protect older people (what "older" means depends on the cost), and do not try to control the epidemic. Consequently, I would suggest rephrasing the abstract or writing ([] means removal): "This strategy consists in rapidly intervening in older populations [to protect the older people during the initial phase of the epidemic and (if the cost is intermediate or low) to control the epidemic], before progressively alleviating this control. Interventions in the younger population can occur later if the cost associated with the intervention is low. This intervention [targeted at younger people] aims at suppressing the epidemic instead of [] protecting the [older people]."
Answer: We thank the reviewer for this excellent reformulation of our results and the detailed suggestion!
↪ *We rewrote the Author Summary accordingly.*

Introduction – paragraph starting line 11 Optimal control (OC) is presented in a too restrictive (or simplified) way for journal as technical as PLoS Comp. Biol. OC is not restricted to Pontryagin's Maximum Principle and "open-loop" control (meaning that the control variable depends only on time) with a finite time horizon. OC can also accommodate infinite horizon and state-feedback control, using Hamilton-Jacobi-Bellman equations for instance. Consequently, I would suggest rephrasing the introduction or writing ([] means removal): "Things become [] more challenging when the intervention parameter value is a function of time. Optimal control theory [8], [] specifically addresses this issue by identifying a function of time such that [] some criterion is optimised. This has allowed studies to

identify optimal non-pharmaceutical interventions to control infectious diseases such as influenza and COVID-19 [9-12]." [...] "Accounting for two dimensions, time and host age, make the optimisation procedure [much] more challenging because [optimal control theory is usually] applied to ordinary differential equations (ODEs) -something very common- while here we are working on partial differential equations (PDEs) -which is less common, and [much] more challenging.

Answer: Again, we thank the reviewer for the detailed suggestion. We heartily agree that a such 'simplification' in presenting optimal control theory is not necessary for the journal. (In our defense, we had the feeling that other reviewers were not familiar at all with OC.)

↪ *We rewrote this part of the introduction following your suggestion (page 2, lines 11-15 and 23-27).*

Line 68: please place here the sentence from current line 106. "Note that p likely depends on age, but because is it totally unknown, we assume it is a constant."

Answer: ↪ *We replaced the sentence as suggested (line 71).*

Remove "The time since infection grows linearly with time, according to the derivative with respect to i."

Answer: ↪ *We made the change (line 92).*

Fig. 7's legend: "France" should be replaced with "Vietnam".

Answer: We are not sure we understand the concern because Figure 7 is about France.

Line 352: unclear what "despite their same proportion of infected individuals" means, as the lines cannot be distinguished in Fig. 8(e). Same concern with the last part of the sentence re. Fig. 8(d). This echoes a general comment: please make sure that every color can be seen on the graphs.

Answer: We apologize for the lack of clarity. We rewrote the sentence as follows: 'However, the optimal control performs at least 50% better than the uniform control (whether it concerns the whole population or its younger fraction). This result holds despite the nearly identical proportions of infections in the population under 60 years of age.'

↪ *See lines 351-354*

Line 375: remove "shorter".

Answer: ↪ *Done, line 375*

Line 381: replace "extends" with "applies" - see my earlier comments relative to the Author summary. By the way, if my interpretation makes sense, I would suggest updating that part of the Discussion as well.

Answer: We rewrote this part of the discussion in accordance with changes made in the Author Summary. We also replaced 'extends' by 'applies' as suggested.

↪ *See lines 375-383*

Fig. S6: why did you consider the proportion of paucisymptomatic infections p in [0, 0.5] while it was in [0, 0.95] in the sensitivity analysis (Table 1)? I would find it more convincing to show the optimal strategies for larger values of p.

Answer: Thank you for the suggestion. We added a column with a larger value of p in Figure S6. We also updated the comment concerning this figure accordingly.

↪ *See lines 398-401*
